# The impact of the centralized volume-based procurement policy on Chinese pharmaceutical manufacturing firms’ R&D investment: A difference-in-differences approach

**DOI:** 10.1371/journal.pone.0315811

**Published:** 2025-01-03

**Authors:** Fangjun Qiu, Shouming Chen, Yujia Li, Xianjing Wang, Mengfei Zhu

**Affiliations:** School of Economics and Management, Tongji University, Shanghai, China; Tianjin University, CHINA

## Abstract

Centralized drug procurement is a common practice worldwide to relieve the healthcare burden and promote high-quality development in the pharmaceutical industry. However, scholars have not yet reached an agreement on whether centralized procurement can facilitate the innovation activities of pharmaceutical firms. China’s centralized volume-based procurement (CVBP) implemented in 2018 provides an ideal quasi-natural experiment to evaluate the effect of centralized procurement on the R&D investment of the firms. Drawing data from listed manufacturing firms in China’s A-share market (2015–2020), the results from a difference-in-differences analysis with different model specifications indicate that the CVBP significantly promotes the pharmaceutical manufacturing firms’ R&D investment. Moreover, the positive effect of the CVBP on R&D investment is stronger in pharmaceutical manufacturing firms with high marketing expenses. A series of robustness tests including the parallel trend test, placebo test, and the PSM-DID analysis show that our findings are solid. This paper advances our understanding of centralized procurement in emerging markets and provides new insights into how governments and pharmaceutical manufacturing firms can strengthen innovation.

## 1 Introduction

Centralized procurement is a group drug purchasing model guided by the government, an alliance of medical institutions, or a third party. It is designed to regulate the procurement and sale of drugs, to reduce the social burden of medical costs, and to optimize the industry competition pattern [1]. Due to the fundamental role of medical innovation in a country’s sustainable development, many Western countries adopt centralized procurement to address the problem of difficult and expensive access to healthcare and to facilitate demand-oriented innovation in the pharmaceutical industry [2].

“Marketing over R&D” is a long-standing issue in China’s pharmaceutical market, which results in inflated drug prices and a lack of innovation capability in pharmaceutical firms. To ameliorate these problems, China began to explore the shift from decentralized to centralized drug bidding and purchasing in 2000. In more than a decade of continuous practice, the provincial centralized procurement framework associated with the combination of procurement and tendering, quantity and price linkage, and the two-invoice system has taken shape [3]. In November 2018, the General Office of the State Council officially released the “4 + 7” centralized volume-based procurement (CVBP hereinafter) policy, the core of which is centralized purchasing using the “volume for price” strategy. Four municipalities and seven major cities were selected as pilot cities, and the policy was gradually expanded nationwide. Increasingly sophisticated procurement mechanisms, fair bidding processes, and transparent platform supervision significantly save the medical insurance fund and accelerate the survival of the fittest pharmaceutical firms [4, 5].

In previous studies, the impact of centralized procurement on innovation has mostly been explained from a macro perspective, viewing centralized procurement as changes in market demand, government regulation, and public policy [6]. In this study, we aim to observe whether the impact of centralized procurement policies on innovation in China, an emerging economy, is consistent with that in Western countries. Additionally, we shift our focus to the micro level and specifically concentrate on pharmaceutical firms to explore the impact of the CVBP policy on innovation. Many scholars have discussed the positive impact of CVBP in China, finding that the price of generic medicines dropped dramatically after the introduction of the CVBP, and the pharmaceutical manufacturing industry has transformed [7–9]. Despite the prevalence of such topics, theoretical interpretations and empirical research on CVBP regarding its evolving processes and influence on firm strategic decisions remain scarce. Moreover, as a technology-intensive industry, pharmaceutical innovation capability reflects both firm competence and the development level of a country. China has been developing into a powerful country in pharma through original innovation and R&D in generic drugs [10], providing us with a unique context for understanding the effect of centralized procurement on firm innovation in developing countries. Building on this context, we will focus on the following two questions: First, does CVBP promote the R&D investment of pharmaceutical manufacturing firms? Second, if CVBP promotes pharmaceutical R&D investment, does the marketing expenditure on which firms rely to gain profits affect this promotion?

To answer the above questions, we take listed manufacturing firms in China’s A-share market as research samples and employ the difference-in-differences (DID) methodology to test the influence of the CVBP policy on the pharmaceutical manufacturing firms’ R&D investment. Moreover, we group the samples according to their marketing characteristics to investigate the heterogeneous effect of CVBP on R&D investment of firms with heavy or light marketing expenses.

This paper makes contributions to three aspects. First, from the perspective of governmental policy, we expand the research on institutional factors influencing the innovation of pharmaceutical manufacturing firms [11]. Taking the implementation of CVBP as a quasi-natural experiment, we investigate whether centralized procurement forces pharmaceutical firms to invest more in R&D. Second, our paper discusses situations in which centralized procurement promotes pharmaceutical innovation. We aimed to discover the positive moderating effect of marketing expenses and the results reveal the changes in business focus from marketing to the innovation of pharmaceutical manufacturing firms under CVBP [7]. Third, our paper is based on the context of China, which enriches the literature on worldwide changes in the pharmaceutical industry. Given that China is making a transition from a major pharmaceutical country to a world pharmaceutical power and has an institutional context very different from western countries, research on the influence of pharmaceutical policy reform in China is of high value [12, 13]. Fourth, this study examines the impact of CVBP policy on firm innovation from a micro perspective. This discovery underscores the public sector’s role in guiding firm research, driving societal technological progress and innovation. In addition, we hope that our paper can be a reference for pharmaceutical firms and provide some insights on policy improvement for the government as well.

## 2 Background, theory, and hypotheses

### 2.1 Background of the centralized procurement policy

Controlling healthcare spending and promoting the development of the pharmaceutical industry are important economic and social issues worldwide. One general solution is centralized procurement [14, 15]. There are two main international procurement modes, including government direct procurement, such as in the UK and India, and third-party-led procurement, such as GPOs in the US. China has been experimenting with centralized drug procurement practices since the 1990s, and through continuous improvement and changes, a government-led, province-based, integrated procurement model with quantity–price linkage has gradually emerged [3].

In November 2018, the Central Committee for Comprehensively Deepening Reform approved the Pilot Program of Centralized Drug Procurement, identifying four municipalities (Beijing, Shanghai, Tianjin, and Chongqing) and seven main cities (Guangzhou, Shenzhen, Shenyang, Dalian, Xi’an, Chengdu, and Xiamen) as a pilot in the first round. The whole procurement process is conducted on a centralized platform under a national unified standard [16]. The core of this policy is the trade-off between larger procurement quantities and lower prices. First, the public medical institution submits an agreed procurement list of drugs and volumes to the National Health Security Bureau based on 60% to 70% of the estimation of the previous year’s drug consumption. Second, the National Health and Family Planning Commission organizes bidding and price negotiations in which firms that pass the drug consistency evaluation can participate. Finally, for each type of drug, the firms offering the lowest price with the agreed volume win the bid.

In December 2018, the National Joint Purchasing Office announced the first batch of selected drugs, including 22 generic drugs and three proprietary drugs. This practice has significantly reduced drug prices by an average of 52%. In September 2019, the policy was extended to the whole country, and the prices of 25 selected drugs were further reduced by 25%. Up to now, China has completed the fifth batch of CVBP, with the number of selected firms and types of drugs further expanded; 61 drugs were successfully procured with an average price reduction of 56%.

### 2.2 Centralized procurement and pharmaceutical firm innovation

Previous literature has three interpretive perspectives on the topic of the impact of centralized procurement on innovation. Firstly, the government has expressed the importance of technology through the demand from centralized procurement [17]. Secondly, centralized procurement serves as a means for the government to regulate the market [18]. Thirdly, centralized procurement functions as a kind of public policy to improve innovation [19]. Most studies argue that centralized procurement can promote innovation from these three perspectives. However, the question of whether centralized procurement fosters innovation in pharmaceutical firms lacks a consensus. Empirical studies have shown contradictory results in different countries and regions, which suggests that the effect of centralized procurement may depend on the institutional environment to a large degree (20). Some scholars insist that centralized procurement promotes innovation [8, 20, 21], while others suggest that centralized procurement stifles innovation [1, 22].

The structure and regulatory model of the Chinese pharmaceutical market differ significantly from that of the West, especially since China is a latecomer to centralized procurement and innovation [10]. Nevertheless, literature regarding the effect of centralized procurement on the innovation of pharmaceutical manufacturing firms in China is mostly limited to theoretical explanations. A case study from Shenzhen found that, after CVBP intervention, the volume and price of alternative medicines increased [4]. An event study on the innovation investment of pharmaceutical firms found that CVBP rendered less firm value loss for firms with high R&D investment intensity [7]. Research on profit changes has found that price competition caused by centralized procurement decreases firm profits [8]. Hence, the generic pharmaceutical firms should increase R&D and invent original drugs to acquire competitive advantages [5].

### 2.3 Hypothesis development

#### 2.3.1 The CVBP policy in promoting R&D investment

Pharmaceutical firms in China generally have a marketing-driven rather than an innovation-driven value creation model. Fundamentally, this is because under the same circumstances, putting more resources into marketing can create more value for firms than into innovation. The new institutional economics suggests that the incentive structures provided by institutions affect the firm’s decision making, such as innovation based on cost–benefit calculations [23]. As a formal government policy, the CVBP policy forces pharmaceutical firms to increase R&D investment through intensifying market competition, weakening marketing channels, and highlighting R&D shortcomings.

First, reduced profitability associated with increased market competition forces pharmaceutical firms to gain heterogeneous competitive resources through R&D. CVBP successfully alleviated inflated prices of generic drugs by adopting a centralized volume-for-price approach, which crowds out the profits of drug sales while also strengthening market competition [1, 24]. Under such circumstances, cost advantage is the key to winning a centralized bid, so pharmaceutical firms should increase R&D investment to reduce unit production costs and form economies of scale. In addition, the CVBP policy mainly focuses on generic drugs, squeezing the space for generics while dividing the market for innovative drugs [7]. Therefore, pharmaceutical firms are supposed to develop innovative drugs to avoid competing with homogeneous generics and consequently obtaining excessive monopoly profits in the long run [25].

Second, weakened marketing channels make pharmaceutical firms more willing to expand upstream in the value chain. Innovation and marketing are two important value-creation paths for pharmaceutical manufacturing firms. Innovation is expansion up the value chain, while marketing is expansion down the value chain [26]. After the implementation of CVBP, the platform construction and process supervision at the provincial level promote fair competition and inhibit the “secondary bargaining” phenomenon [27], which will directly eliminate the “grey space” of commercial bribery and prompts firms to expand up the value chain by increasing R&D investment.

Third, highlighted R&D shortcomings and external preference for innovation make firms invest more in innovation. CVBP requires that only medicines that passed consistency evaluation can participate in centralized bidding, which not only makes demands on the pharmaceutical firms’ own drug quality and innovation capabilities but also makes external stakeholders more concerned about the firms’ R&D investment [28, 29]. Therefore, we propose Hypothesis 1.

*H1*: The CVBP policy will positively affect the pharmaceutical manufacturing firms’ R&D investment.

#### 2.3.2 The influence of marketing expenses

As discussed above, CVBP has largely intensified competition in the pharmaceutical industry and weakened the role of marketing in value creation. In that case, whether a pharmaceutical firm relies on marketing to make a profit may determine the extent to which it suffers from CVBP [30]. Specifically, compared with firms with low marketing expenditure, pharmaceutical manufacturing firms with heavy marketing expenses rely more on marketing to win purchase orders and expand market share. However, the essence of the CVBP policy is price competition, which greatly reduces the price of medicine and compresses the cost of circulation and marketing. In this case, only firms with cost advantages have the ability to continue to participate in the volume-based procurement. Therefore, pharmaceutical manufacturing firms that originally relied heavily on marketing must increase R&D investment to acquire heterogeneous competition resources to avoid being eliminated by the market.

On the other hand, CVBP decreases marketing costs, such as market maintenance, drug marketing, and advertising. For firms with high marketing expenses, this process significantly reduces the occupation of resources, which allows pharmaceutical manufacturing firms to spare more resources on the new drug R&D, main product modification, and talent introduction. Therefore, we propose Hypothesis 2.

*H2*: Compared to pharmaceutical firms with low marketing expenses, the positive effect of CVBP is stronger for pharmaceutical manufacturing firms with high marketing expenses.

## 3 Data and method

Using the data of 1521 manufacturing firms listed in mainland China from the year 2015 to 2020, we compare the R&D input of pharmaceutical manufacturing firms and other manufacturing firms to investigate how the R&D investment of Chinese pharmaceutical manufacturing firms is influenced by the CVBP policy.

### 3.1 Research method

The DID method is a widely employed research approach to objectively evaluate the effects of policies [31]. In the field of pharmaceutical policy evaluation, compared with traditional pharmaceutical policy evaluation methods such as the Interrupted Time Series method, the DID method is more suitable to investigate year-term policy influences, which contributes to its popularity in management research. The DID method takes the exogenous policy as the main influencing factor to construct a quasi-natural experiment. The samples are divided into the treated group affected by the exogenous variable and the control group not affected. By studying the differences between the dependent variable value of the treated group and the control group, we can explore the influence of the policy [32]. By controlling all main variables except the policy, DID avoids the endogeneity of the policy itself, and excludes the impact of unobservable individual heterogeneity on the results. It also avoids the problems of reverse causality and variable omission in traditional regression research [33]. Thus, DID method can obtain an unbiased estimate of the policy effect.

To accurately analyse the influence of the CVBP policy, we used the DID method and divided our samples into a treated group, which contained pharmaceutical manufacturing firms affected by the policy, and a control group, which contained other manufacturing industries not affected by the policy. In this way, the impacts of unobservable differences among pharmaceutical manufacturing firms on the research results are avoided, and the research conclusion is more accurate and stable.

### 3.2 Model structure

In order to study how the CVBP policy influences the R&D input of Chinese pharmaceutical manufacturing firms, we constructed a model (1) applying the DID method:

$$\begin{array}{c} RDI_{it}=\alpha_{0}+\alpha_{1}post\times treat+\alpha control_{i,t}+\varepsilon_{i,t} \end{array}$$ (1)

where *RDI* represents the R&D investment intensity of the firm. *Post* is a dummy variable indicating whether an observation is observed after the implementation of the CVBP policy. *Treat* is a binary variable that denotes membership in the treated group. *Control* represents the control variables. This study focuses on the coefficients of the interaction term of *post* and *treat*, *α*_1_. If the coefficient *α*_1_ is significantly positive, it indicates that the R&D input of Chinese pharmaceutical manufacturing firms is encouraged by the enforcement of the CVBP, which is consistent with the hypotheses of this study.

### 3.3 Variable description

#### 3.3.1 Dependent variable

R&D intensity is the dependent variable of our research and is named RDI. R&D intensity is measured by the rate of annual R&D investment to sales revenue, which is a common indicator of firm R&D investment and has been applied in many studies on firm R&D activities [34–36].

#### 3.3.2 Independent variables

The dummy variables, *post* and *treat*, are the independent variables of our research. Together they indicate whether the observation is a pharmaceutical firm observed after the introduction of the CVBP policy.

*Post* indicates whether the samples were observed after the CVBP policy. The CVBP policy was nationally approved on November 14th, 2018, at the 5th meeting of the Central Committee of Comprehensively Deepening Reform. Since panel data is used in the research, and the countrywide introduction of the CVBP policy took place mainly in 2019 and the end of 2018, we assign the dummy variable post to the observation samples in 2019 and 2020 to 1 in the regression. We choose the post event window up to 2020 because the second wave of centralized volume-based procurement (CVBP) begun in March 2020. The year 2018 and the previous years are considered to be before the implementation of the policy.

*Treat* indicates whether the sample is affected by the policy. If the sample belongs to the treated group, treat equals 1, otherwise it equals 0. As the CVBP policy only influences pharmaceutical firms, we took pharmaceutical manufacturing firms as the treated group while taking other manufacturing firms as the control group. Simultaneously, we performed a parallel trend analysis to validate the appropriateness of the selection of the treated group and the control group. We followed the industry classification of the China Securities Regulatory Commission in 2012. The samples with the industry code of “C27” were chosen as the treated group. The manufacturing industry firms except for those belonging to the pharmaceutical manufacturing industry were chosen as the control group, and their classification code began with “C”.

#### 3.3.3 Control variables

Drawing upon prior studies investigating the R&D of firm, we included the firm size, firm age, financial situation, firm value, and ownership as control variables. By choosing these control variables, we excluded the possible influence of these factors on the R&D intensity. Good business performance helps firms have more confidence to participate in innovation investment [37]. ROA and Tobin’s Q are controlled by the ratio of net profit to total assets and the ratio of market value to asset value, respectively. The leverage level of firms will have an impact on innovation, and high debt is not conducive to the investment in innovation,so debt to asset ratio is controlled [38]. Large firms have an advantage in R&D because they can apply the results to a larger output, thus spreading the cost of R&D [39]. Hence, we controlled firm size through the logarithm of the value of the total asset. Previous study found that younger firms are exposed to more risk when it comes to innovation, while older firms have more predictable return on their innovation efforts [40]. Therefore, we controlled firm age using the operating life of the firm. State-owned enterprises can obtain the support of the government when seeking innovation resources, so that they can carry out innovation activities more smoothly [41]. Thus, firm ownership was controlled with 1 for state-owned enterprises and 0 for non-state-owned enterprises. Meanwhile, concentrated ownership can enhance innovative activities as it ensures pursuit of shareholder interests. However, excessive concentration may lead to agency problems, favoring large shareholders’ interests over the firm’s innovative investments, potentially harming small shareholders and impeding economic benefits [41]. Here, we controlled largest shareholder’s share as a proxy of ownership concentration. Lastly, firms with separated ownership and control rights will respond to policies slower than those with integrated ownership and control rights [42]. Therefore, this paper controlled the separation between ownership and control rights with the difference between the share controlled and owned by the actual controller.

Descriptions of the control variables along with the dependent variable and independent variables are given in detail in Table 1:

**Table 1:** Variable description.

Variable	Variable Name	Description
Dependent variable		
R&D intensity	RDI	R&D input/Sales revenue
Independent variables		
Treated group	treat	Equals 1 if the observation belongs to the pharmaceutical industry (coded C27 in Securities Supervision Commission Industry Code), otherwise, it equals 0
Policy influence	post	Equals 1 if a firm is observed on or after 2018, otherwise, it equals 0
Moderating variable
Marketing intensity	mktex	Marketing expense/Sales revenue
Control variables		
ROA	NROA	Net profit/Total asset
Tobin’s Q	tobin’s	Market value/Asset value
Debt to asset ratio	lev	Debt/Asset
Firm size	lgsize	The logarithm of the value of the total asset
Firm age	age	Operating life of the firm
Firm ownership	owner	Equals 1 if the firm is a state-owned enterprise, otherwise, it equals 0
Largest shareholder’s share	big1	The share that the largest shareholder owns
The separation between ownership and control rights	sepe	The difference between the share controlled and owned by the actual controller

### 3.4 Data source

Taking the listed manufacturing firms in China’s A-share market as research samples, and taking 2015–2020 as the research period, we obtained a total of 8450 observations of 1521 enterprises, including 161 pharmaceutical manufacturing firms (899 observations), and 1360 other manufacturing firms (7551 observations). In the process of sample screening, the following exclusion criteria were used: (1) Special Treatment (ST) and *ST firms at risk were excluded; (2) Samples listed after 2015 were excluded; (3) Samples with incomplete variable data were eliminated. The R&D intensity, firm size, firm age, financial status, and ownership applied in this study were all based on the CSMAR database (China Stock Market and Accounting Research Database). The CSMAR database is mostly used in Chinese research and is highly recognized.

## 4 Results

By conducting descriptive statistics analysis and correlation analysis, we obtained an initial understanding of our data and prepared for the regression analysis. The regression results verify our hypotheses, and the robust tests make our conclusions more credible.

### 4.1 Descriptive statistics analysis

The observation numbers, mean value, standard deviation, maximum values, and minimum values of all research variables of all samples are listed in Table 2. Our study used 8450 observations. The average R&D intensity is 4.500, indicating that all manufacturing firms spend 4.5% of their revenue on R&D activities on average. Among all manufacturing firms, 161 firms focused on pharmaceutical manufacturing, and these pharmaceutical manufacturing firms were taken as the treated group. Furthermore, we compared the variable differences between the treated group and the control group in the pre and post period of CVBP policy, and the results are presented in Table 3. The table shows that the RDI of the treated group is significantly higher than that of the control group, and almost all control variables exhibit significant differences, indicating that the selection of these control variables is necessary.

**Table 2:** Descriptive statistics analysis: “Marketing over R&D”.

Variables	Number	Mean Value	Standard Deviation	Min	Max
RDI	8450	4.5	4.136	0	88.56
post	8450	0.348	0.476	0	1
treat	8450	0.106	0.308	0	1
NROA	8450	0.0384	0.0745	-1.125	0.816
tobin’s	8450	2.184	1.764	0.684	92.25
lev	8450	0.408	0.19	0.00836	2.29
lgsize	8450	22.31	1.161	17.81	27.55
age	8450	18.68	5.371	6	53
owner	8450	0.318	0.466	0	1
big1	8450	32.76	13.77	2.87	89.09
sepe	8450	4.931	7.744	-7.640	56.11

**Table 3:** Comparison between the treated and the control group.

**Panel A: pre-period**						
	Treatment		Control			
Variables	N	Mean	N	Mean	MeanDiff	p-Value
RDI	466	5.92	3943	4.509	1.41	0
NROA	466	0.047	3943	0.031	0.016	0
tobin’s	466	2.292	3943	1.794	0.498	0
lev	466	0.331	3943	0.43	-0.098	0
lgsize	466	22.321	3943	22.446	-0.125	0.03
age	466	20.867	3943	20.056	0.811	0.001
owner	466	0.249	3943	0.336	-0.087	0
big1	466	31.94	3943	31.707	0.233	0.725
sepe	466	5.749	3943	4.829	0.92	0.016
**Panel B: post-period**						
	Treatment		Control			
Variables	N	Mean	N	Mean	MeanDiff	p-Value
RDI	433	4.784	3608	4.273	0.511	0.02
NROA	433	0.076	3608	0.041	0.036	0
tobin’s	433	3.016	3608	2.496	0.52	0
lev	433	0.31	3608	0.405	-0.095	0
lgsize	433	22.079	3608	22.186	-0.106	0.065
age	433	17.723	3608	17.018	0.705	0.007
owner	433	0.229	3608	0.317	-0.088	0
big1	433	33.723	3608	33.904	-0.181	0.799
sepe	433	5.712	3608	4.843	0.87	0.027

### 4.2 Correlation analysis

Table 4 presents the correlation coefficients among the variables involved in this study. There is no correlation coefficient greater than 0.5, which means there is no serious collinearity problem and the variables are reasonably selected. The correlation coefficient between the interaction term of *treat* and *post* and the dependent variable *RDI* is 0.084, and the coefficient demonstrates statistical significance at a significance level of 1%, indicating that the pharmaceutical manufacturing firms make R&D investments after the new procurement policy’s implementation, which is consistent with our hypotheses.

**Table 4:** Correlation analysis.

**Variable**	**RDI**	Treat*Post	**NROA**	**Tobin’s**	**Lev**	**Lgsize**	**Age**	**Owner**	**Big1**	**Sepe**
RDI	1									
treat*post	0.084 ***	1								
NROA	-0.077 ***	0.011	1							
tobin’s	0.114 ***	0.031 ***	0.173 ***	1						
lev	-0.167 ***	-0.074 ***	-0.335 ***	-0.233 ***	1					
lgsize	-0.154 ***	0.005	0.059 ***	-0.328 ***	0.476 ***	1				
age	-0.099 ***	0.099 ***	-0.025 **	-0.056 ***	0.106 ***	0.129 ***	1			
owner	-0.110 ***	-0.027 **	-0.070 ***	-0.084 ***	0.261 ***	0.310 ***	0.259 ***	1		
big1	-0.084 ***	-0.020 *	0.139 ***	0.013	0.023 **	0.152 ***	-0.020 *	0.165 ***	1	
sepe	-0.057 ***	0.019 *	0.055 ***	-0.024 **	0.081 ***	0.142 ***	0.051 ***	0.021 **	0.188 ***	1

*p < 0.1,

**p < 0.05,

***p < 0.01.

### 4.3 Main effect

Table 5 lists the regression results of model (1) under random effect, individual fixed effect, and both individual and time fixed effect. Considering the influence of COVID-19 on the pharmaceutical industry in 2020, we controlled the annual differences through time-fixed effects to obtain a fairer policy impact. Sub-model 1 does not control individual and annual differences. Sub-model 2 controls individual differences, and sub-model 3 controls both individual and annual differences. Treat*post reflects the R&D intensity differences between pharmaceutical manufacturing firms and other manufacturing firms before and after the CVBP policy. In the three sub-models, the regression coefficients of the variable treat*post are all positive and significant at the significance level of 1% (*α* = 1.7191, *p* < 0.01; *α* = 1.0035, *p* < 0.01; *α* = 0.9015, *p* < 0.01), which means that after the implementation of the CVBP policy, pharmaceutical manufacturing firms have invested more in R&D than other manufacturing firms. That is, the R&D investment of pharmaceutical manufacturing firms was promoted by the CVBP policy. The results support Hypothesis 1.

**Table 5:** DID analyses of the CVBP policy’s influence on the R&D investments of pharmaceutical manufacturing firms.

Variable	-1	-2	-3
	RDI	RDI	RDI
treat*post	1.7179 ***	1.0035 ***	0.9015 ***
	-0.233	-0.323	-0.329
NROA	8.5257 ***	6.1476 ***	6.0702 ***
	-0.665	-1.223	-1.219
age	0.0553 ***	0.0559 *	0.045
	-0.008	-0.031	-0.032
tobin’s	0.2246 ***	0.0813 **	0.1174 **
	-0.023	-0.041	-0.047
lev	3.5440***	0.7334	0.8014
	-0.289	-1.095	-1.089
lgsize	0.0381	0.1893	0.1662
	-0.049	-0.399	-0.395
owner	0.2780 ***	0.5934	0.5298
	-0.103	-0.398	-0.396
big1	0.0171 ***	0.0038	0.0037
	-0.003	-0.009	-0.009
sepe	0.0087	0.0001	-0.0003
	-0.006	-0.018	-0.018
_cons	6.5199 ***	8.0906	8.041
	-1.052	-8.627	-8.602
Fixed effect	No	Yes	Yes
Year dummies	No	No	Yes
Observations	8450	8450	8450
*R* ^2^	0.068	0.04	0.043

Standard errors in parentheses;

*p < 0.1,

**p < 0.05,

***p < 0.01.

### 4.4 The moderating effect of marketing expenses

To further investigate the business transformation of pharmaceutical manufacturing firms through the new procurement policy and verify Hypothesis 2, we grouped the samples according to their marketing characteristics and investigated how the CVBP policy influenced the R&D investment in pharmaceutical manufacturing firms with high and low levels of marketing expenses. Specifically, firms with marketing intensities higher than the 25th percentile of the marketing intensities in the industry are considered as heavy marketing firms. The industry percentiles are often used in heterogeneity analyses [43, 44]. The difference in influences of the CVBP policy on R&D input of heavy and light marketing firms reveals the changes in pharmaceutical manufacturing firms’ business decisions in the face of the new policy.

Based on the discussions in Hypothesis 2, we grouped the samples into the heavy marketing group and the light marketing group by comparing the marketing intensity of each firm with the average annual industry marketing intensity. For both the heavy marketing firms and the light marketing firms, we carried out the DID analysis. The individual and annual differences were controlled and Table 6 displays the results of the DID analyses conducted in both groups. In the heavy marketing group, the coefficient of treat*post is significantly positive (*α* = 1.8967, *p* < 0.01), while in the light marketing group, the coefficient of treat*post is not significant. It shows that for heavy marketing pharmaceutical manufacturing firms, the CVBP policy significantly encouraged their R&D activities, while light marketing pharmaceutical manufacturing firms did not show significant growth in R&D activities after the policy intervention, which is consistent with Hypothesis 2.

**Table 6:** DID analyses of heavy marketing and light marketing firms.

Variable	Heavy Marketing Firms	Light Marketing Firms
	RDI	RDI
treat*post	1.8967 ***	-0.0885
	-0.652	-0.167
NROA	-10.2455 ***	-3.6117 ***
	-2.406	-0.679
age	-0.0232	0.0584 *
	-0.073	-0.032
tobin’s	-0.0933 *	-0.0952 *
	-0.055	-0.055
lgsize	0.8927	0.0021
	-0.569	-0.131
lev	-1.2635	-1.5929 ***
	-1.929	-0.427
owner	-0.1182	0.4984 **
	-0.377	-0.207
big1	0.0263 *	-0.0059
	-0.014	-0.007
sepe	0.0203	0.0061
	-0.022	-0.01
_cons	-13.4737	3.8763
	-11.875	-2.845
Fixed effect	Yes	Yes
Year dummies	Yes	Yes
Observations	3226	5224
*R* ^2^	0.082	0.053

Standard errors in parentheses;

*p < 0.1,

**p < 0.05,

***p < 0.01.

### 4.5 Heterogeneity analysis of firm size

Large firms are less resource crunched and one would believe that the CVBP policy will have a greater effect on smaller firms. We conduct a heterogeneity analysis of firm size to test the assumption, the sample are grouped according to the median of firm size and divided into small and large firms for regression. The results in Table 7 show that the change of large firms after the policy is similar with small firms, which is inconsistent with the expectation. The possible reason is that a higher proportion of large pharmaceutical listed companies in China are generic manufacturers, while a higher proportion of smaller firms are innovators. Innovator companies invest heavily in R&D to develop new drugs, while generic manufacturers focus on producing low-cost versions of existing medications which face more severe resource constraints. Relatively, Generic manufacturers face more severe resource constraints compared with the Innovators.

**Table 7:** DID analyses of large size and small size firms.

Variables	Large Size Firms	Small Size Firms
	RDI	RDI
did	0.8026*	0.9611*
	-0.426	-0.546
NROA3	-4.2752*	-6.8178***
	-2.371	-1.234
age	0.1898**	0.0085
	-0.078	-0.059
tobin3	-0.1283**	-0.0861
	-0.062	-0.06
lgsize	-1.4491	-0.0311
	-1.135	-0.3
lev	0.7992	-1.9208**
	-2.465	-0.749
owner	0.5420**	0.6934
	-0.264	-0.767
big1	-0.0104	0.0018
	-0.018	-0.011
sepe	-0.0104	0.0069
	-0.031	-0.018
_cons	34.3036	6.4817
	-24.361	-6.212
Fixed Effect	Yes	Yes
Year dummies	Yes	Yes
N	4273	4177
*R* ^2^	0.055	0.056
Coefficient difference	0.729	
p-value		

Standard errors in parentheses;

*p < 0.1,

**p < 0.05,

***p < 0.01.

The coefficient difference *p*-value is calculated by chow-test

### 4.6 Robustness checks

#### 4.6.1 Parallel trend test

**Fig 1:**
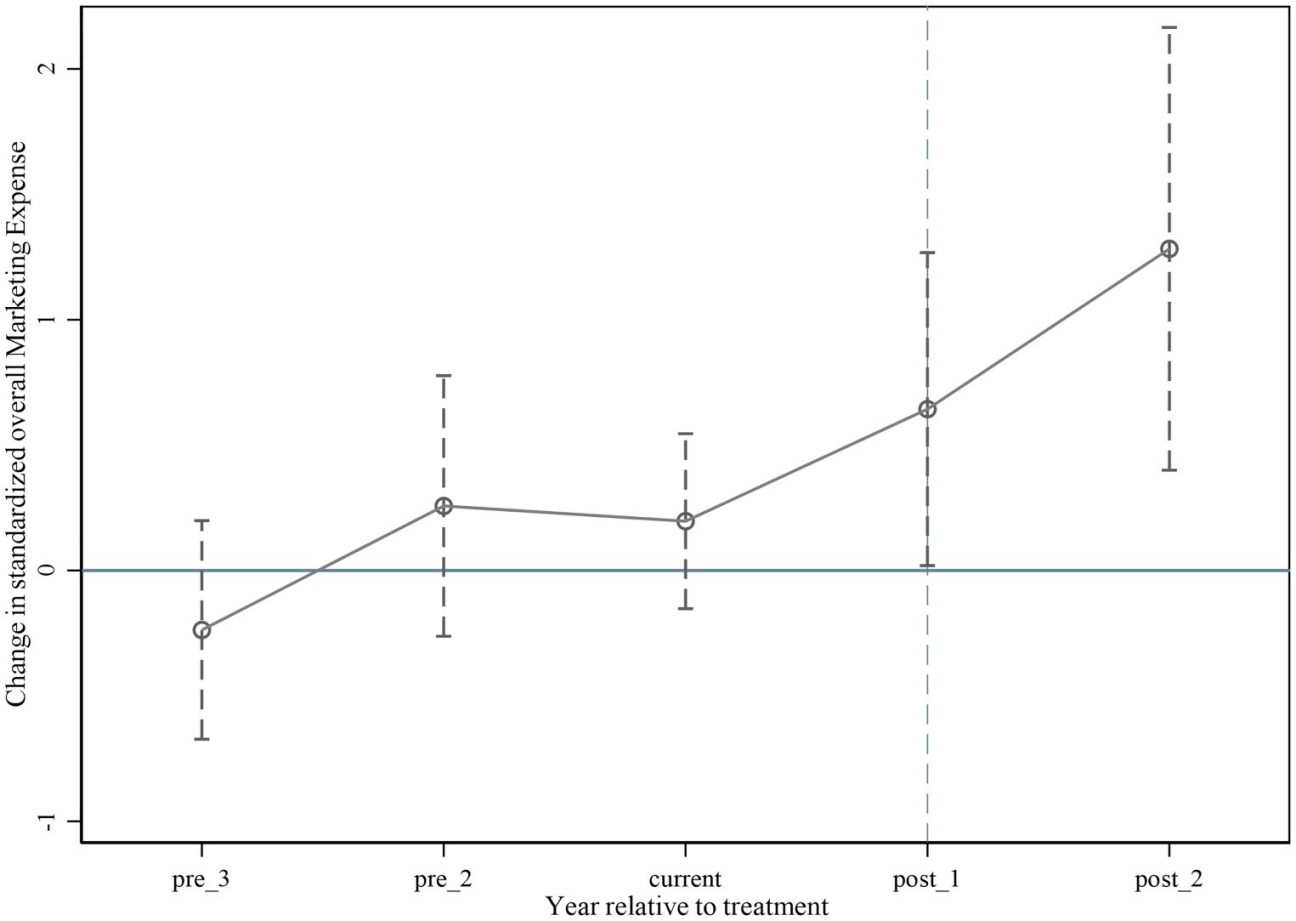
Parallel trend test.

The parallel trend test was carried out to verify that the treated group shared a similar R&D input trend with the control group. We reveal the influence of the CVBP policy on the R&D investment of pharmaceutical manufacturing firms by comparing the differences in the trend of R&D investment of pharmaceutical manufacturing firms and other manufacturing firms. To ensure that this difference was caused only by the policy, it is necessary to examine whether the R&D intensity of the treated group and the control group share similar trends before being impacted by the CVBP policy. The results of the parallel trend test are shown in Fig 1. *Pre*_3, *pre*_2, *current*, *post*_1 and, *post*_2 indicate the three years before the policy, the year of policy implementation, one year, and two years after the policy, respectively. The regression coefficients are not significant in the three years before the policy implementation, but the coefficients for 1 year (*α* = 0.8508, *p* < 0.01; *α* = 0.6434, *p* < 0.01) and 2 years (*α* = 1.4996, *p* < 0.01; *α* = 1.2825, *p* < 0.01) after policy implementation are significantly positive. The regression results revealed that the R&D input of the treated group and the control group maintained a similar trend in 2018 and before, but differed afterwards. The difference is the net effect of the CVBP policy, which means that this policy encouraged pharmaceutical manufacturing firms to invest more in R&D.

#### 4.6.2 Placebo test

The placebo test can further investigate whether the promotion effect of the CVBP policy on R&D inputs of pharmaceutical manufacturing firms is caused by other factors. We construct a randomly picked treated group by repeating random sampling. By studying the changes in R&D intensity of the random “treated groups”, rather than the pharmaceutical manufacturing firms, we can investigate whether the research results are stable. Statistics show there are 161 pharmaceutical manufacturing firms in the whole research sample. Therefore, we randomly selected 161 firms in the whole sample as the treated group and took the remaining firms as the random control group to repeat 500 random sampling experiments to explore the difference in R&D investment between the random treated group and the random control group.

**Fig 2:**
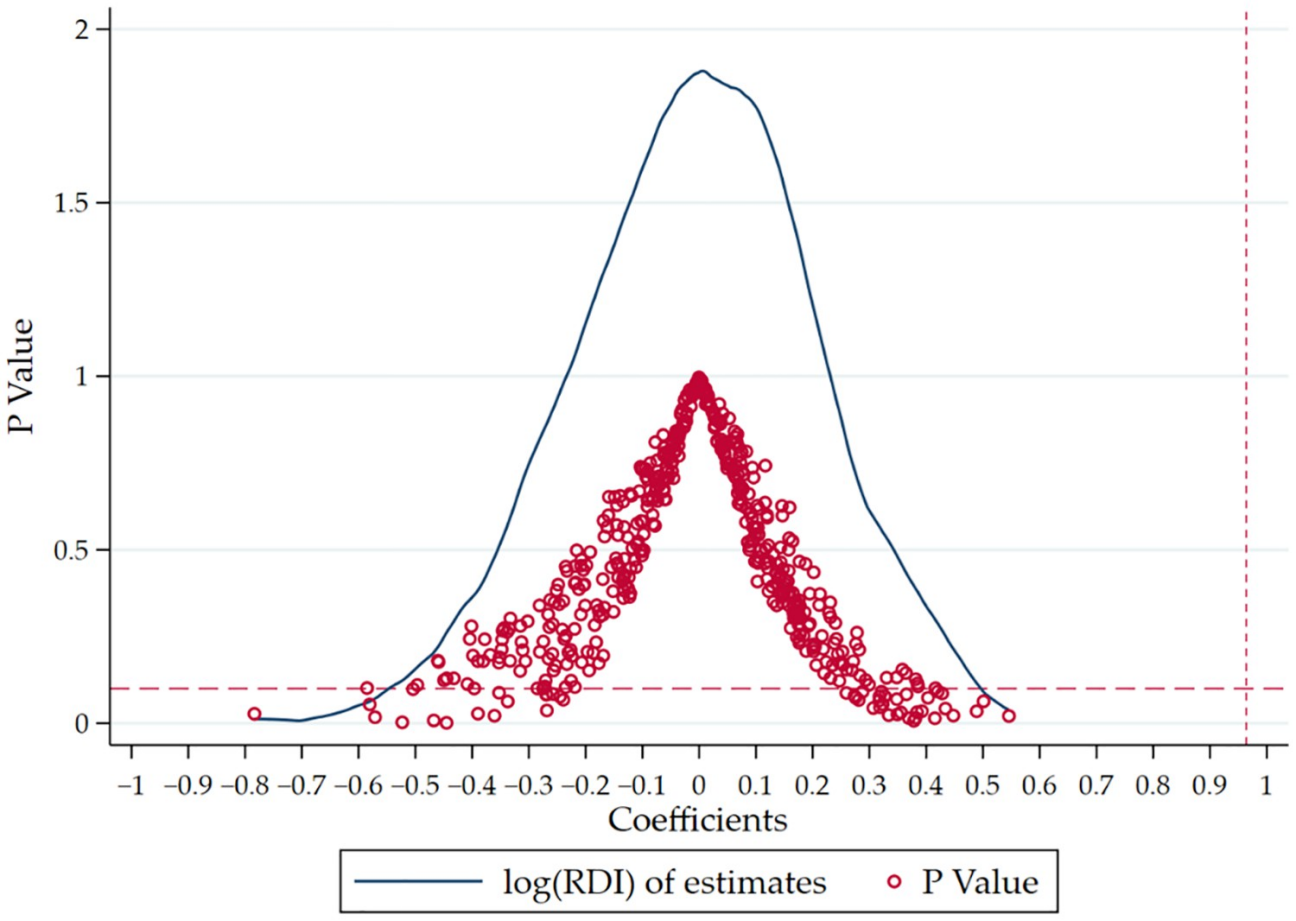
Placebo test.

As shown in Fig 2, the points in the figure represent the regression *p*-value of each random sampling. The horizontal red dotted line represents the significance level of 10%, and the p-values of most regression results fall above the significance level of 10%, which means that most effects of the 500 random sampling experiments cannot prove that the treated group has a higher R&D investment level than the control group after 2018. In contrast, pharmaceutical manufacturing firms, as the treated group, have significantly increased their R&D intensity after the CVBP policy came to influence in 2018, which further proves that the R&D promotion effect of CVBP policy on the pharmaceutical manufacturing firms is not caused by other random factors.

#### 4.6.3 PSM-DID analysis

The PSM-DID (PSM—Propensity Score Matching) method matches the pharmaceutical manufacturing firms in the treated group with other manufacturing firms, to construct a more standard treated group and control group and to make the research results more accurate. We conducted the year-wise kernel matching using the pre-event characteristics. For each sample in the treated group, the sample from controlled group was matched based on control variables [45]. Table 8 shows the matching results. The mean values of the control variables of the treated group and the control group are close, indicating that the treated group and the control group share similar characteristics in all control variables and that the matching makes sense.

**Table 8:** PSM results.

Variables	Mean		%Bias	*t*-test		V(T)/V C
	Treated	Control		t	*p* > |*t*|	
NROA	0.04486	0.04283	2.4	0.21	0.835	1.04
age	21.929	21.835	1.9	0.16	0.874	0.76
tobin’s	2.614	2.6894	-3	-0.27	0.791	0.68 *
lgsize	22.362	22.389	-2.5	-0.22	0.827	0.59 *
lev	0.34142	0.35874	-9.9	-0.89	0.375	0.96
owner	0.25974	0.27369	-3	-0.28	0.783	/
big1	31.076	31.187	-0.9	-0.07	0.941	0.82
sepe	5.6054	5.4021	2.6	0.22	0.825	0.95

The matched DID regression outcomes are presented in Table 9 Sub-model 2 included an annual fixed effect based on sub-model 1. In the two models, the coefficients of the variable treat*post are significantly positive (*α* = 0.8599, *p* < 0.05; *α* = 0.7988, *p* < 0.05). This further shows that the catalytic role of the CVBP policy on the R&D input of pharmaceutical manufacturing firms is significant.

**Table 9:** PSM-DID regression results.

Variables	-1	-2
	RDI	RDI
treat*post	0.8930***	0.7845**
	-0.319	-0.324
NROA	-7.6081***	-7.5099***
	-1.173	-1.173
age	0.0632**	0.0441
	-0.028	-0.032
tobin’s	-0.0759**	-0.1165***
	-0.038	-0.044
lgsize	-0.2347	-0.2017
	-0.441	-0.438
lev	-1.2613	-1.3484
	-1.107	-1.101
owner	0.6213	0.5573
	-0.392	-0.39
big1	0.0069	0.0067
	-0.009	-0.009
sepe	-0.0017	-0.0024
	-0.019	-0.019
_cons	9.1255	9.0471
	-9.457	-9.436
Year dummies	No	Yes
Observations	8277	8277
*R* ^2^	0.055	0.058

Standard errors in parentheses;

*p < 0.1,

**p < 0.05,

***p < 0.01.

## 5 Conclusions

Based on the Chinese background, our study delved into the influence of the CVBP policy on R&D investment in pharmaceutical manufacturing firms. By using the DID method, we found that the CVBP policy increased the R&D input of pharmaceutical manufacturing firms. Furthermore, we examined how the policy influenced the R&D of firms with different marketing expenses, and found that pharmaceutical manufacturing firms that spent more on marketing were more significantly encouraged to invest in R&D by the CVBP policy. Finally, the results of the parallel trend test and placebo test ensured the reliability of our conclusions.

This study explored the key role of the CVBP policy in the changes in the innovation of pharmaceutical manufacturing firms, which was consistent with previous conclusions [6, 46, 47]. Drawing upon innovation models that highlight the importance of demand, previous studies have proposed that public procurement can serve as a potent instrument for fostering innovation and catalyzing technological advancement. These series of studies explained the impact of centralized procurement on innovation from a macro perspective [19]. However, this study takes a micro perspective and explores the impact of the CVBP policy on the marketing activities of pharmaceutical companies, and subsequently its impact on innovation, enriching the research on the impact mechanism of centralized procurement. Considering the specific context of China and the pharmaceutical industry, our study indicated that the new drug procurement policy crowded out the profit margin of pharmaceutical firms through intensifying market competition and weakening marketing roles, thus prompting firms to increase technology research and development to acquire a competitive advantage. Clearly, the essence of the CVBP policy lay in forcing the R&D of pharmaceutical firms [48]. Our findings appeared to reaffirm this view once again.

Our findings provide implications for both business organizations and the government. For firms, the managers should be sensitive to the regulatory atmosphere and be aware of any regulatory risk, as the government plays an especially important role in all industries in China. The CVBP policy intensified competition in the industry. Increasing R&D investment is important for firms to maintain sustainable development in face of competition. When the market is filled with homogeneous, cheap, and low-R&D drugs, drugs with more innovation show better resilience. On the other hand, both marketing activity and innovation activity can create value. However, when the new policy reduces the profit space of marketing, firms should adjust marketing strategies by optimizing the allocation of resources.

We also provide information for policymakers. The CVBP policy has effectively altered the business focus of Chinese pharmaceutical manufacturing firms. However, many pharmaceutical firms suffered severe losses during the introduction of this policy. Drug R&D is expensive, time-consuming, and too difficult for small and medium-sized pharmaceutical firms. The government should offer extra support in R&D activity to those firms which cannot afford further R&D activities and be aware of the oligarchic trend in the industry. The fact is that there is still a huge gap between China and developed countries in drug R&D, and the gap cannot be bridged in one day. Besides reforming the industry through policies, the government should take a broader perspective and be more forward-thinking by also paying attention to other segments of innovation in the pharmaceutical manufacturing industry such as education, talent attraction, and hardware development. Meanwhile, the experience in China also has the implication for emerging markets that policymakers can accelerate the development of key sectors for national economic growth through centralized procurement.

Limitations exist in the process of discussing the impact of the CVBP policy on pharmaceutical manufacturing firms pointing out further research possibilities for us. This study takes all pharmaceutical firms as samples without further breaking down their types. However, the pharmaceutical firms in China can be classified into generic pharmaceutical business and proprietary pharmaceutical business, and these two types of businesses may have different responses to the CVBP policy. Studying the differences in R&D activities of generic pharmaceutical firms and proprietary pharmaceutical firms will bring more contributions. The CVBP policy aims to narrow the profit of generic drugs and to stop pharmaceutical manufacturing firms from totally relying on making generic drugs but not innovations. The study of these two types of pharmaceutical manufacturing firms will better reveal and test the actual effect of the CVBP policy. However, we found that the pharmaceutical manufacturing firms which focus on proprietary drugs were too rare to study using empirical research methods. A case study on a proprietary-drug-oriented pharmaceutical manufacturing firm may provide more implications for the CVBP policy on pharmaceutical manufacturing firms.

## Supporting information

S1 Dataset (XLS)
